# Estimation and Short-Term Prediction of the Course of the HIV Epidemic Using Demographic and Health Survey Methodology-Like Data

**DOI:** 10.1371/journal.pone.0130387

**Published:** 2015-06-19

**Authors:** Stéphanie Blaizot, Benjamin Riche, David Maman, Irene Mukui, Beatrice Kirubi, Jean-François Etard, René Ecochard

**Affiliations:** 1 Service de Biostatistique, Hospices Civils de Lyon, F-69003, Lyon, France; 2 Université de Lyon, F-69000, Lyon, France; 3 Université Lyon 1, F-69100, Villeurbanne, France; 4 CNRS UMR 5558, Equipe Biostatistique-Santé, Laboratoire de Biométrie et Biologie Evolutive, F-69100, Villeurbanne, France; 5 Epicentre, F-75011, Paris, France; 6 National AIDS and STDs Control Program, Nairobi, Kenya; 7 Médecins Sans Frontières, Nairobi, Kenya; 8 UMI 233 TransVIHMI, Institut de Recherche pour le Développement, INSERM U1175, Université Montpellier 1, F-34000, Montpellier, France; The University of Melbourne, AUSTRALIA

## Abstract

**Background:**

Mathematical models have played important roles in the understanding of epidemics and in the study of the impacts of various behavioral or medical measures. However, modeling accurately the future spread of an epidemic requires context-specific parameters that are difficult to estimate because of lack of data. Our objective is to propose a methodology to estimate context-specific parameters using Demographic and Health Survey (DHS)-like data that can be used in mathematical modeling of short-term HIV spreading.

**Methods and Findings:**

The model splits the population according to sex, age, HIV status, and antiretroviral treatment status. To estimate context-specific parameters, we used individuals’ histories included in DHS-like data and a statistical analysis that used decomposition of the Poisson likelihood. To predict the course of the HIV epidemic, sex- and age-specific differential equations were used. This approach was applied to recent data from Kenya. The approach allowed the estimation of several key epidemiological parameters. Women had a higher infection rate than men and the highest infection rate in the youngest age groups (15–24 and 25–34 years) whereas men had the highest infection rate in age group 25–34 years. The immunosuppression rates were similar between age groups. The treatment rate was the highest in age group 35–59 years in both sexes. The results showed that, within the 15–24 year age group, increasing male circumcision coverage and antiretroviral therapy coverage at CD4 ≤ 350/mm^3^ over the current 70% could have short-term impacts.

**Conclusions:**

The study succeeded in estimating the model parameters using DHS-like data rather than literature data. The analysis provides a framework for using the same data for estimation and prediction, which can improve the validity of context-specific predictions and help designing HIV prevention campaigns.

## Introduction

Mathematical models have played important roles in the understanding of epidemics and in the study of the impacts of various behavioral or medical measures [1–3]. Numerous models with various complexities and assumptions have been developed to provide long-term predictions of the future courses of the HIV epidemic, especially the impact of various prevention and treatment strategies [4–7]. In these models, the parameters are often taken from studies carried out in other distant countries with different contexts, which may result in biased results and misguided policies.

Demographic and Health Surveys (DHS) are “nationally-representative household surveys that provide data for a wide range of monitoring and impact evaluation indicators in the areas of population, health, and nutrition” and, since 2001, include HIV testing [8]. In many countries, including Sub-Saharan Africa, these surveys have provided precise estimates of national HIV prevalence in the adult population.

In the present paper, we propose a modeling approach for short-term predictions of the spread of the HIV epidemic. This approach includes an estimation of model parameters from survey data conducted with the DHS methodology. The mathematical model we propose for prediction takes into account sex, age, the natural progression of HIV infection through different stages, the use of antiretroviral therapy (ART), and the ageing of the population.

To illustrate our purpose, this approach was applied to a recently designed household survey that used the DHS methodology: the Ndhiwa HIV Impact in Population Survey (NHIPS). We also modeled the impacts of hypothetical prevention and treatment interventions such as increasing ART coverage among eligible HIV-infected individuals, increasing the proportion of medically circumcised HIV-uninfected men, and implementing pre-exposure prophylaxis in HIV-uninfected women.

## Methods

### The NHIPS

The NHIPS is a district-representative cross-sectional population survey conducted in September-November 2012 [9,10]. It used the DHS methodology to provide information regarding the HIV epidemic in the adult population (15–59 years old) in the district of Ndhiwa (Nyanza Province, Kenya). The NHIPS consisted of a household questionnaire (completed with the head of the household) plus an individual questionnaire and laboratory tests (HIV test, CD4 count, and viral load). The survey selected randomly 165 clusters of 20 households. The 3,300 successfully interviewed households included 16,198 persons (8,493 women and 7,705 men), of whom 6,833 were eligible and 6,076 agreed to participate.

The primary objective of the NHIPS was to estimate the HIV incidence using incidence assays. The secondary objectives included the determination of HIV prevalence, the proportion of HIV-positive respondents in need for ART, the ART coverage, the proportion of HIV-positive respondents with undetectable viral load, the HIV testing and counseling coverage, the proportion of medically circumcised men, and the access to prevention of mother-to-child transmission (PMTCT) services.

From the NHIPS data, we used variables age, sex, HIV status, CD4 cell count, and the following self-reported variables: circumcision status (aided by drawings of penises), date of the most recent HIV test and its result, date of first positive HIV test, and the ART status with the date of ART initiation.

Ethics permissions were obtained from the KEMRI Ethical Review Committee (number 347) and the Comité de Protection des Personnes “Ile-de-France XI” (Saint-Germain-en-Laye, France; CPP number 12056). All participants in the study provided written informed consent. For individuals under the age of 18 years never married and never having lived in a consensual union, a specific mentor permission with written consent was obtained.

### Epidemiological model

**Fig 1:**
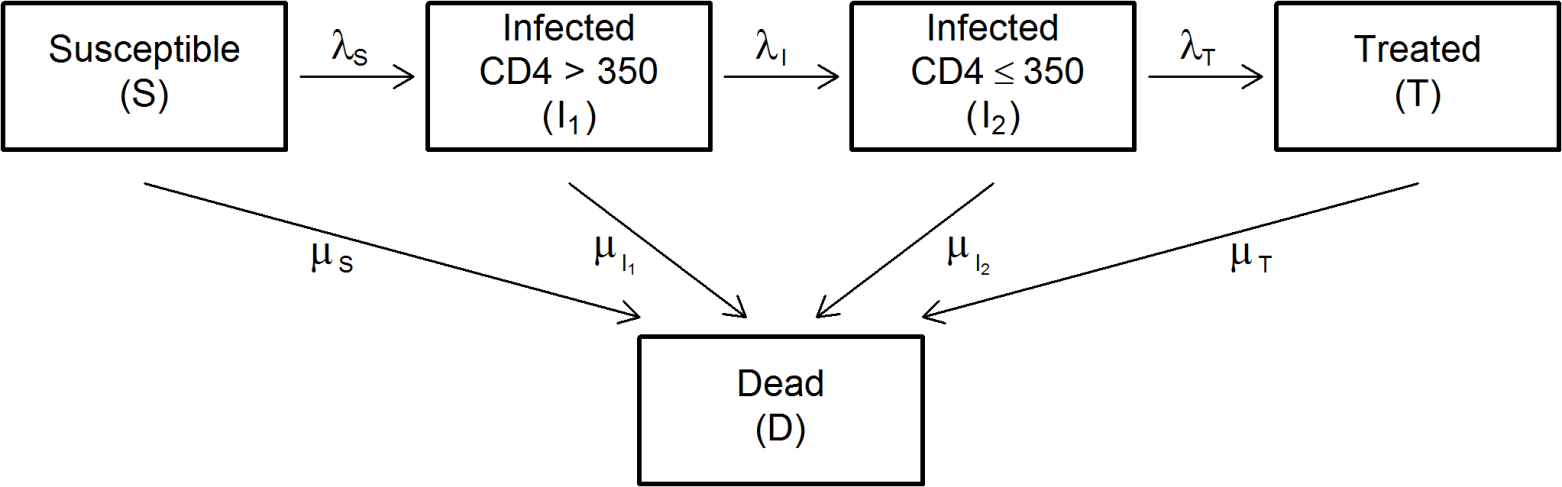
Schematic representation of the HIV model. The boxes represent the model compartments. The arrows represent the flows (or transitions) between compartments. The model splits the population into five groups: susceptible individuals (S), HIV-positive individuals (not on ART) with ≤ 350 CD4 cells/mm^3^ (I_1_), HIV-positive individuals (not on ART) with > 350 CD4 cells/mm^3^ (I_2_), HIV-positive individuals on ART (T), deceased individuals (D). *λ*_*S*_ denotes the force of infection, *λ*_*I*_ the immunosuppression rate, *λ*_*T*_ the treatment rate, and *μ* the compartment-specific mortality rate. All the compartments are sex- and age-specific.

The model, shown in Fig 1, describes HIV transmission, the untreated-disease progression, and ART use in a heterosexual population. It splits the population into compartments according to sex, age, and HIV status. The population of infected individuals was split into three compartments according to the CD4 cell count and the ART status: 1) Compartment I_1_: untreated HIV-positive individuals with a CD4 cell count > 350 cells/mm^3^; 2) Compartment I_2_: untreated HIV-positive individuals with a CD4 cell count ≤ 350 cells/mm^3^ (immunosuppressed individuals); and, 3) Compartment T: HIV-positive individuals under ART. An additional Compartment S was dedicated to HIV-negative (or susceptible) individuals.

The model parameters are the following: i) the force of infection *(λ*_*S*_ *)*; ii) the "immunosuppression rate" *(λ*_*I*_ *)*; i.e., the rate at which an individual moves from > 350 to ≤ 350 cells/mm^3^ CD4 cell count; iii) the "treatment rate" *(λ*_*T*_ *)* or the ART initiation rate; and, iv) the mortality rate *(μ)*.

### Estimation and prediction approach

To predict the course of the HIV epidemic in the adult population, our approach included an estimation step and a prediction step.

#### Estimation step

Estimating the model parameters included three stages. First, we used the HIV status, the self-reported ART status, and the CD4 cell count at the time of the survey to assign each individual to one of the above-cited compartments.

Second, we derived individuals’ states (as described by the compartments) during the past year from the individuals’ histories (self-reported dates of first positive HIV test, last HIV test and its result, and ART initiation). We only derived the previous year to minimize the recall bias and avoid making too strong assumptions about individuals’ histories. More precisely, HIV-negative individuals at the date of the survey were considered as previously HIV-negative. Depending on the date of the first positive HIV test and/or the result of the last HIV test, untreated HIV-positive individuals with CD4 cell counts > 350 cells/mm^3^ (Compartment I_1_) were considered to have been already HIV-positive (positive test result) or HIV-negative (negative test result) one year before the survey. In untreated HIV-positive individuals with CD4 cell counts ≤ 350 cells/mm^3^ at the moment of the survey (Compartment I_2_), the status regarding the CD4 cell count one year before was obtained by applying a 15% CD4 cell count reduction per year [11]. Depending on the date of ART initiation, treated individuals were considered to have been already treated one year preceding the survey or untreated HIV-positive with CD4 cell counts ≤ 350 cells/mm^3^ one year preceding the survey. Logical or probabilistic rules were applied when the retrospective information was incomplete or lacking (6.3% and 1.2% of all cases, respectively). The logical rules consisted in the following assumptions: i) HIV-positive individuals under a treatment initiated within the previous year were considered to have been HIV-positive one year preceding the survey; ii) untreated HIV-positive individuals with low CD4 cell counts were considered to have been already HIV-positive one year preceding the survey; iii) untreated HIV-positive individuals with high CD4 cell counts but no information about previous testing were considered to have been HIV-positive at least one month preceding the survey.

Third, we calculated the number of transitions between pairs of compartments and the time spent by each individual in each compartment.

A sensitivity analysis regarding the assumption of the CD4 cell count decline was performed assuming a 10% decline per year [12] as well as a 20% decline per year [13].

#### Prediction step

In this step, the compartment sizes (i.e., the number of individuals in each compartment) stemming from the NHIPS survey (applied on the 2009 Ndhiwa District census) and the parameters provided by the estimation step were used to predict the short-term course of the HIV epidemic in the adult population (15–59 years old).

To illustrate our approach, we modeled four scenarios for the short-term impacts (duration of simulation of three years, start 2012—end 2015).

The first scenario was “no change in the current interventions” in which all the rates were set to their estimated values and kept unchanged over the whole duration of the simulation.

The second scenario was an increase in the ART coverage under the current guidelines: this scenario was designed to explore the impacts of an increase in ART coverage using the current CD4 cell count threshold for ART initiation (350 cells/mm^3^) on the HIV prevalence and incidence rate. This increase in ART coverage aims to increase the proportion of individuals living with HIV but with a low viral load, which would reduce infectiousness. We assumed that the ART coverage among eligible individuals (i.e., the number of treated individuals divided by the sum treated plus immunosuppressed untreated individuals) would increase linearly from 70% in 2012 to 90% in 2015. By reducing the viral load, ART is assumed to reduce infectiousness by 96% [14].

The third scenario was an increase in the medical male circumcision coverage. We assumed that 60% of HIV-negative men would be circumcised at the end of the duration of the simulation with a linear increase over that duration. The baseline proportion of HIV-negative circumcised men was set at 25% (NHIPS Survey, 2012). The protective effect of circumcision was assumed to reduce female-male transmission by 60% [15–18].

The fourth scenario was the use of pre-exposure prophylaxis (PrEP) in women. We assumed that 20% of HIV-negative women would be using it at the end of the duration of the simulation with a linear increase over that duration. The protective effect of PrEP was assumed to reduce male-female transmission by 50%.

#### Inference methods

In the estimation step, we used a statistical method based on the likelihood decomposition [19,20]. In this method, each state is considered separately and the transitions from any given state to another may be considered as concurrent risks. The number of transitions between two states is modeled as a realization of a Poisson process. The likelihood of the complete model is decomposed into several conditionally independent likelihoods. This decomposition is feasible whenever there are no interactions between the individual courses. Here, the probability of infection for an uninfected individual depends on the prevalence. At this step, the period being about one year, the change in the prevalence was nearly negligible. Thus, the force of infection was not split into prevalence and a transmission coefficient (this interaction between susceptible and infected individuals will be taken into account in the prediction step through a frequency-dependent force of infection [21]). We estimated the model parameters, in men and women, in three age groups (15–24, 25–34, 35–59 years) and using a stratification by six “Divisions” (residence areas). The likelihood was maximized using the Newton-Raphson option of PROC NLP of SAS 9.2 software and the confidence intervals were calculated with Wald method (see details in S1 Text).

Because of the low number of deaths recorded in the NHIPS, the above statistical method could not be used; instead, Poisson regressions were used to estimate AIDS-related and AIDS-unrelated mortality rates using overall mortality rates from the Kenyan DHS [22], NHIPS-external but local data on the proportion of AIDS-related deaths [23,24], and the HIV prevalence observed in the NHIPS (see details in S1 Text). We assumed that S, I_1_, and T individuals had the same risk of death whereas I_2_ individuals had an additional risk of death due to AIDS.

In the prediction step, we formulated the mathematical model as a system of sex- and age-specific differential equations (see details in S1 Text). The differential equations were written for each one-year age class. The compartment sizes were calculated by one-year age classes but the parameter values were estimated by age classes (15–24, 25–34, and 35–59 years) for the force of infection, the immunosuppression rate, and the treatment rate, or by five-year age classes for the mortality rates.

## Results

### Estimation

Table 1 shows that, in women, the infection rate was higher in the youngest age groups: 47 and 48 per 1000 person-years (PY) in age groups 15–24 and 25–34 years *vs*. 26 in age group 35–59 years. In men, the infection rate was the highest in age group 25–34 years (41 per 1000 PY *vs*. 9 and 27 in age groups 15–24 and 35–59 years).

**Table 1:** Estimated transition rates and their 95% confidence intervals (per 1000 person-years).

Parameter	Women	Men
Infection rate *(λ*_*S*_ *)*		
15–24 years	47 [35–63]	9 [4–19]
25–34 years	48 [33–69]	41 [24–67]
35–59 years	26 [17–40]	27 [16–46]
Immunosuppression rate *(λ*_*I*_ *)*		
15–24 years	153 [75–312]	0*
25–34 years	207 [132–323]	335 [170–660]
35–59 years	198 [116–338]	216 [113–412]
Treatment rate *(λ*_*T*_ *)*		
15–24 years	439 [236–815]	519 [61–4440]
25–34 years	480 [337–683]	334 [169–659]
35–59 years	793 [576–1092]	631 [461–863]

* Value estimated at 0 because no transition between compartments I_1_ and I_2_ was observed in this group.

The immunosuppression rate was similar between age groups be it in men or in women (from 153 to 335 per 1000 PY). In other words, the time until CD4 cell count drop below 350 cells/mm^3^ ranged from 3 to 7 years depending on the age group. In men aged 15–24 years, the immunosuppression rate was estimated at 0 because no transition to ≤ 350 CD4 cells/mm^3^ was observed in the data.

The treatment rate was the highest in age group 35–59 years in men (631 per 1000 PY *vs*. 519 and 334 in age groups 15–24 and 25–34 years) and in women (793 per 1000 PY *vs*. 439 and 480 in the age groups 15–24 and 25–34 years). In other words, the time until ART initiation ranged from 1 to 3 years depending on the age group. In men aged 15–24 years, the 95% confidence interval was quite large because few men were infected and, subsequently, eligible for treatment or actually treated.

Using a 10% or a 20% CD4 cell count decline per year, the immunosuppression rate (per 1000 PY) in women ranged from 107 [45–257] to 167 [85–329] in age group 15–24, from 185 [114–298] to 241 [160–361] in age group 25–34, and from 156 [84–290] to 217 [131–360] in age group 35–59. In men, the immunosuppression rate was 0 in age group 15–24 and ranged from 312 [152–637] to 376 [202–699] in age group 25–34 and from 184 [90–375] to 286 [167–488] in age group 35–59. Changing the CD4 count decline resulted in very slight changes in the treatment rate and in no changes in the infection rate.

Table 2 shows the estimates of the mortality rates stratified by sex, age group, and CD4-cell-count categories. As expected, the mortality rates increased with age, were slightly higher in men than in women, and higher in individuals with low CD4 cell counts.

**Table 2:** Estimated mortality rates and their 95% confidence intervals (per 1000 person-years).

Parameter	Women	Men
> 350 CD4 cells/mm^3^ *(μ*_*S*,_ *μ*_*I1*,_ *μ*_*T*_ *)*		
15–19 years	1.45 [0.97–1.93]	2.31 [1.68–2.93]
20–24 years	2.90 [2.27–3.53]	2.92 [2.26–3.58]
25–29 years	4.81 [4.00–5.62]	3.58 [2.86–4.30]
30–34 years	4.69 [3.89–5.48]	6.61 [5.50–7.72]
35–39 years	6.75 [5.59–7.90]	6.69 [5.49–7.90]
40–44 years	8.18 [6.61–9.74]	11.03 [9.01–13.05]
45–59 years	8.70 [6.42–10.98]	13.42 [10.55–16.29]
≤ 350 CD4 cells/mm^3^ *(μ*_*I2*_ *)*		
15–19 years	9.38 [6.27–12.48]	27.39 [19.94–34.83]
20–24 years	9.67 [7.58–11.76]	13.31 [10.32–16.30]
25–29 years	15.16 [12.60–17.73]	12.23 [9.77–14.69]
30–34 years	25.45 [21.14–29.76]	14.53 [12.09–16.97]
35–39 years	32.10 [26.60–37.60]	22.85 [18.73–26.98]
40–44 years	28.74 [23.24–34.24]	32.62 [26.63–38.61]
45–59 years	32.44 [23.94–40.94]	48.56 [38.17–58.94]

Mortality rates were estimated using a Poisson model, Kenyan DHS data [22], and external data on the proportion of AIDS-related deaths [23,24].

### Prediction

**Fig 2:**
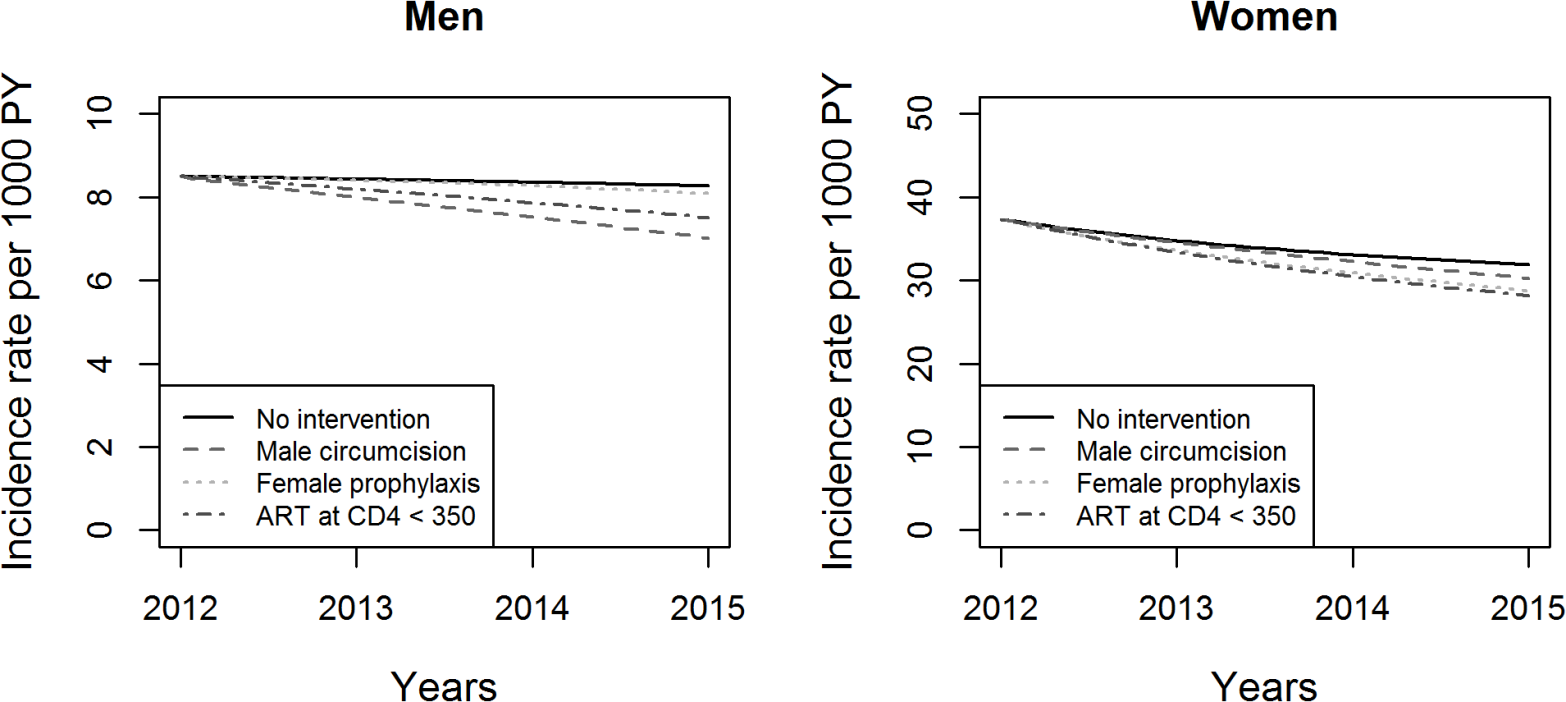
Changes in the HIV incidence rate over the time of simulation in men and women aged 15–24 years. The Y-axes have uneven ranges of values for better legibility.

Fig 2 shows the change in the HIV incidence rate in age class 15–24 years over the time of simulation with each of the four scenarios in men and women. In this age group, on the very short term (3 years), circumcision in men (60% coverage and 60% effectiveness) would have the highest impact on the incidence rate. In women, increasing the ART coverage at the threshold 350 CD4 cells/mm^3^ would have the highest impact followed by pre-exposure prophylaxis (20% coverage and 50% effectiveness). Given the already high (70%) baseline ART coverage at threshold 350 CD4 cells/mm^3^, increasing ART coverage would have, on the short term, a limited impact on HIV prevalence and incidence rate.

**Fig 3:**
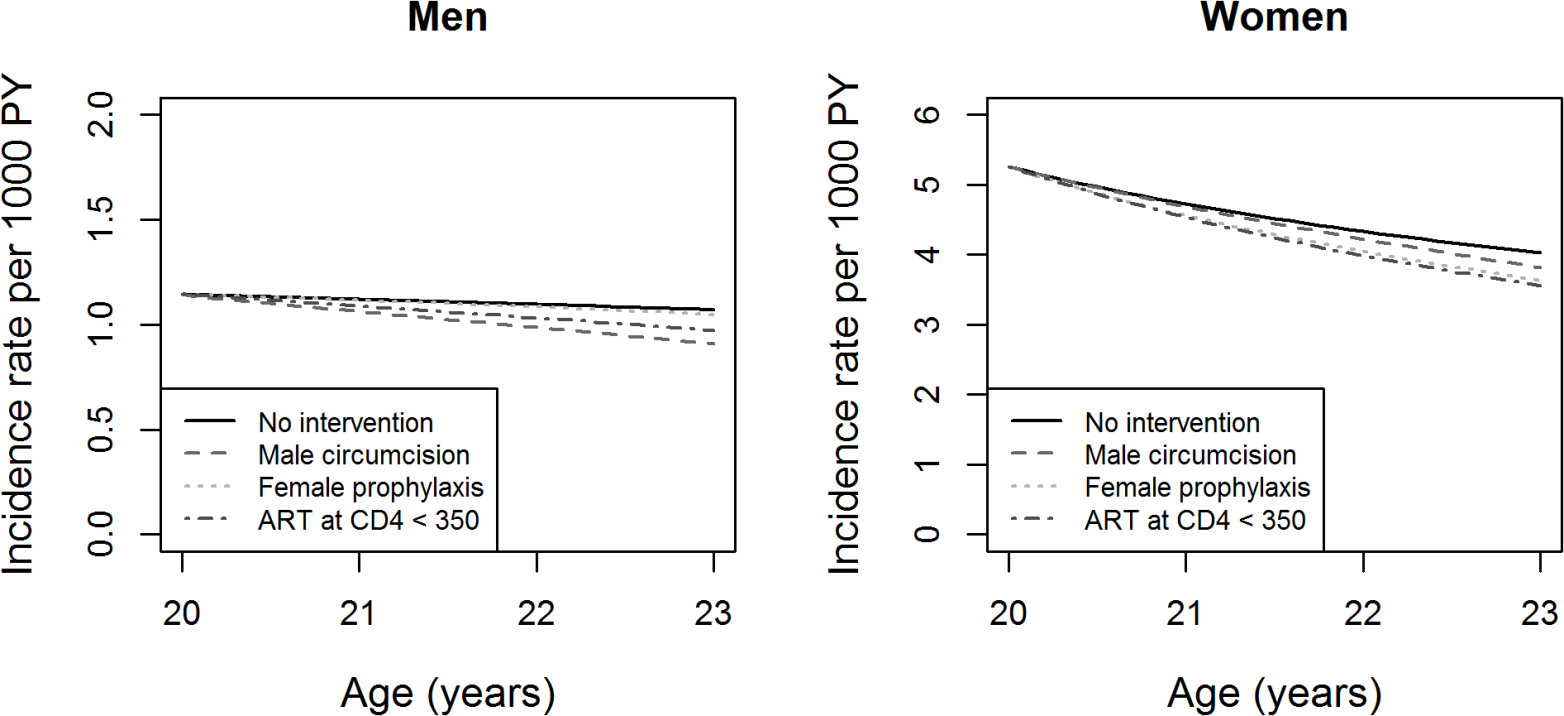
Changes in HIV incidence rate over the time of simulation in men and women aged 20 years in 2012. The Y-axes have uneven ranges of values for better legibility.

Fig 3 shows the change in the HIV incidence rate over time in men and women aged 20 years in 2012 (this is equivalent to a “follow-up” of this age group over time). The prevalence would slightly increase. Again, circumcision in men (60% coverage and 60% effectiveness) would have the highest impact on the incidence rate. In women, increasing ART coverage at the threshold 350 CD4 cells/mm^3^ would have the highest impact followed by pre-exposure prophylaxis (20% coverage and 50% effectiveness). Here too, given the already high (70%) baseline ART coverage at threshold ≤ 350 CD4 cells/mm^3^, increasing ART coverage would have a limited impact on HIV prevalence and incidence rate.

## Discussion

The study proposed a methodology to estimate context-specific parameters that can be used in mathematical modeling of short-term HIV spreading using Demographic and Health Survey (DHS)-like data.

This approach allowed estimation of several key epidemiological parameters. Indeed, the present results showed that women had a higher infection rate than men and that women had the highest infection rate in the youngest age groups (15–24 and 25–34 years) whereas men had the highest infection rate in age group 25–34 years. These results are consistent with those of other studies in that women acquire the virus at younger ages than men [25].

The immunosuppression rate was similar between age groups be it in men or women. The time until CD4 cell count drop below 350 cells/mm^3^ ranged from 3 to 7 years depending on the age group. This is consistent with studies that estimated this duration to nearly 4 years [26,27]. In men aged 15–24 years, the immunosuppression rate was estimated at 0 because no transition to ≤ 350 CD4 cells/mm^3^ was observed in the data; this is not surprising because men seem to acquire the virus past 25 years and because their CD4 cell counts take time to decrease. Using a 10% and a 20% CD4 cell count decline per year changed only slightly the estimates of the immunosuppression rate; depending on the age group, the time until CD4 cell count drop below 350 cells/mm^3^ ranged, respectively, from 3 to 9 years and from 3 to 6 years.

The treatment rate was the highest in age group 35–59 years in men and women; this may be explained by the fact that individuals in this age group are more likely to have been tested and/or put on treatment than individuals in younger age groups. In the literature, studies that provide treatment rates are scarce because it is difficult to estimate the time during which an individual is eligible. Using data from the Masiphumelele community (South Africa) among individuals with CD4 cell counts below 200 cells/mm^3^ (the South African threshold to start ART at the time of the study), Johnson et al. [28] estimated the ART initiation rates of years 2004 to 2009 to range from 36.5 to 107.5 per 100 PY in men and from 51.9 to 303.1 per 100 PY in women. The results for 2009 were higher than ours; this may be explained by two facts: i) the eligibility criterion was 200 CD4 cells/mm^3^ *vs*. 350 here; and ii) the denominator included only diagnosed and in-care individuals. Here, the denominator of the treatment rates included both diagnosed and undiagnosed individuals eligible for treatment. Therefore, the ART initiation rates here take into account the testing, linkage-to-care, and retain-in-care rates in people eligible for treatment.

As expected, the mortality rates increased with age, were slightly higher in men than in women, and higher in individuals with low (*vs*. high) CD4 cell counts. The high mortality rate in men aged 15–19 years with ≤ 350 CD4 cells/mm^3^ can be explained by the fact that they are generally less subject to other causes of death than older people. Overall all-cause mortality rates were close to those estimated in Northern Malawi [29]. The mortality rates estimated here in individuals > 350 cells/mm^3^ (both HIV-negative and positive individuals) were close to those estimated in other studies [30–32]. The mortality rates estimated in HIV-individuals ≤ 350 cells/mm^3^ were lower here than in other studies [31,32]; this may be explained by the quite high proportion of HIV-positive individuals under ART in the present study. Anyway, comparing mortality rates is difficult because mortality depends on various factors that differ between countries.

Regarding prediction, the approach showed that, in age class 15–24 years and on the short term, circumcision in men, increasing ART coverage, and pre-exposure prophylaxis in women had slight impacts on the incidence rate, though higher in comparison with no change in the current interventions. Here, the baseline ART coverage was very high (70%); therefore, the ART intervention had a limited impact on the HIV incidence rate. The impacts on HIV prevalence are limited to the short term because the prevalence includes past cumulated infections as well as new ones. These results are consistent with those of a recent modeling study that compared ART, circumcision, and behavior change interventions on HIV incidence in the adult population in KwaZulu-Natal (South Africa) [33]. In that study, circumcision and ART at 200 CD4 cells/mm^3^ (the standard threshold to initiate ART before 2011) would have similar effects on the HIV incidence rate over 4 years (which is nearly a 20% reduction from the baseline incidence rate). Two other studies that compared ART and PrEP interventions, in South Africa and more particularly in KwaZulu-Natal, showed that these interventions could have a positive impact on HIV incidence over 10 years [34,35]. However, in most mathematical models used by Eaton et al. [36], ART initiation at 350 CD4 cells/mm^3^ would have a limited impact on incidence over 20 years in South Africa.

One strength of the present approach is the use of the same data for estimation of the model parameters and for prediction. Indeed, the NHIPS population survey includes both the current status (which helps estimating the content of each compartment) and individuals’ histories (which help estimating the transition rates between compartments). NHIPS-external but local data were used to estimate mortality rates, taking into account the HIV prevalence observed in the NHIPS. Moreover, the reduction in CD4 cell count we used stemmed from an external source. The statistical method used to estimate model parameters (using likelihood decomposition) has been widely used in medicine [19,20,37]. Usually, the model parameters are difficult to obtain; they are taken from several, often non-local sources. By using local sources, we expected more accurate predictions than those that use non-local parameters. Actually, the use of local data in prediction models is scarce; two recent studies attempted to use mainly local data to predict the spread of HIV in Malawi and Kenya [38,39].

The present approach may be applied to DHS data. However, contrarily to the NHIPS, DHS data lack information on CD4 cell counts; in the future, DHS could be brought to collect this additional information like in other national surveys [40–42]. Moreover, the complex sampling method (multistage) used for such surveys has to be taken into account (for example, through the design effect). Here, the design effect was not taken into account because its value was almost 1. Indeed, the methodology used a cluster sampling where the number of surveyed individuals was proportional to the number of inhabitants. DHS data provide very useful information that improve the understanding of HIV transmission and its spread. For example, a recent study focused on estimating HIV transmission rates before couple formation, within partnership or extra-couple partnerships by sex and country; it used 23 DHS data from eighteen West-African and Sub-Saharan countries [43]. The authors’ approach, as ours, relied on retrospective reconstruction of individuals’ infection states using self-reported survey data as well as laboratory tests; however, they inferred retrospective states since the beginning of the epidemic in each country whereas we reconstructed only the previous year to minimize the recall bias and avoid making too strong assumptions about the individuals’ histories. Moreover, their approach relied on external estimates of mortality, HIV prevalence, and ART coverage whereas our approach relied on external estimates of mortality, but taking into account the observed HIV prevalence in the NHIPS.

The parameter estimates and the initial compartment sizes, therefore the prediction results, depend on the quality of the data collected. Indeed, in the present paper, these estimates are based on both self-reported information (not further ascertained) and biological data. Misreporting the time elapsed since the last HIV test before the survey and its result may under- or overestimate the force of infection. This approach may be potentially validated using data from prospective cohorts which include demographic information, HIV status, repeated CD4 cell counts, ART status, etc. Alternatively, simulated data may be used to validate the approach, as already done by other authors who studied potential biases in the estimation of HIV acute phase infectivity from the Rakai study [44]. Simulating data allows testing for possible biases (e.g., selection bias or information bias) and robustness of estimators through comparisons between estimates obtained from the simulated data and the chosen parameters. Here, simulation may be used to study, for example, the ranges of the immunosuppression rate estimates in case of heterogeneity in CD4 cell reduction or test misreporting of the date of the last HIV test result. Anyway, a clear understanding and knowledge of most underlying mechanisms are required to simulate data close to realistic data.

A number of models have been already developed in attempts to predict future courses of the HIV epidemic, especially in hyperendemic settings, including various intervention scenarios. These models have diverse complexities; for example, the inclusion of age structure [5], sex-specific compartments [45], co-infections [46], several levels of CD4 cell counts or HIV stages [33,47,48], or several risk groups [45]. They are therefore based on a wide variety of assumptions. The present model accounts for the heterogeneity in HIV prevalence, infection risk, and duration of infection between sexes and age classes, avoiding thus the use of averaged trends. Moreover, when untreated, HIV infection may be considered in two stages according to the CD4 cell count threshold: below or above 350 cells/mm^3^ (i.e., "remote" *vs*. "recent" infection, respectively); this presumes an increased risk of death in individuals with ≤ 350 CD4 cells/mm^3^. Here, we stratified the data by Divisions (residence areas) in the estimation step to obtain robust confidence intervals. It would be also interesting to study heterogeneity in HIV prevalence in smaller geographical units. In our modeling approach, we did not include a Who-Acquires-Infection-From-Whom (WAIFW) matrix because of lack of data. In particular, the higher prevalence in young women than in young men may suggest the absence of assortative age-mixing patterns.

In the present study, the threshold of 350 cells/mm^3^ was considered because it was the current threshold at the time of the survey in Kenya and in several other Sub-Saharan African countries. The 2013 WHO guidelines recommend initiating ART at CD4 ≤ 500 cells/mm^3^ [49]. The adoption of the latter threshold will make it necessary to add to the above-suggested model a flow between Compartments I_1_ and T or to add an intermediate compartment between I_1_ and I_2_.

The model presented here is a simplification of the reality but the methodology it uses for estimation and prediction may be used in more complex models. For example, it is possible to create additional compartments for CD4 cell counts, but sufficient data are required for precise estimates of the model parameters. It is also possible to use information about the viral load by creating compartments based on viral load categories or on both viral load and CD4 cell count categories. In expressing the force of infection, we did not add a coefficient to translate the fact that individuals with low CD4 cell counts have higher viral loads (thus higher infectiousness) than individuals with high counts; indeed, individuals with low CD4 cell counts might be sexually less active [50].

The model may also include individuals who stop their treatment for various reasons (e.g., failure or adverse events) by adding flows between the compartments of treated and untreated individuals (like in [51,52]). We did not consider this option because, in the NHIPS, only two men reported having stopped their treatment.

In conclusion, we propose here a methodology to predict the course of the HIV epidemic through the estimation of model parameters using survey data conducted with the DHS methodology. The approach allows using the same data for estimation and prediction, ensuring thus reliable results. Furthermore, this approach can be adapted to more complex models that capture additional types of information (demographic or clinical). This approach is robust because it relies on few assumptions: the information stem from the data. The scenarios we imagined are mainly illustrations of the proposed approach.

## Supporting Information

S1 Text Technical Appendix. (PDF)

## References

[1] Anderson RM, May RM (1991) Infectious diseases of humans: dynamics and control Oxford: Oxford University Press.

[2] Keeling MJ, Rohani P (2008) Modelling infectious diseases in humans and animals Princeton: Princeton University Press.

[3] Garnett GP, Cousens S, Hallett TB, Steketee R, Walker N (2011) Mathematical models in the evaluation of health programmes. Lancet 378: 515–525. S0140-6736(10)61505-X [pii]; 10.1016/S0140-6736(10)61505-X 21481448

[4] Johnson LF, White PJ (2011) A review of mathematical models of HIV/AIDS interventions and their implications for policy. Sex Transm Infect 87: 629–634. sti.2010.045500 [pii]; 10.1136/sti.2010.045500 21685191

[5] Bacaer N, Pretorius C, Auvert B (2010) An age-structured model for the potential impact of generalized access to antiretrovirals on the South African HIV epidemic. Bull Math Biol 72: 2180–2198. 10.1007/s11538-010-9535-2 20349152

[6] Boily MC, Lowndes CM, Vickerman P, Kumaranayake L, Blanchard J, Moses S, et al (2007) Evaluating large-scale HIV prevention interventions: study design for an integrated mathematical modelling approach. Sex Transm Infect 83: 582–589. sti.2007.027516 [pii]; 10.1136/sti.2007.027516 17942574 PMC2598645

[7] Eaton JW, Johnson LF, Salomon JA, Barnighausen T, Bendavid E, Bershteyn A, et al (2012) HIV treatment as prevention: systematic comparison of mathematical models of the potential impact of antiretroviral therapy on HIV incidence in South Africa. PLoS Med 9: e1001245 10.1371/journal.pmed.1001245PMEDICINE-D-12-00020 [pii]. 22802730 PMC3393664

[8] Measure DHS, ICF International. Available: http://dhsprogram.com/. Accessed August 2014.

[9] Maman D, Mukui I, Kirubi B, Riche B, Zeh C, Masson S, et al. (2013) Good program coverage in high HIV prevalence settings in Western Kenya: preliminary results of the Ndhiwa HIV Impact in Population Study. International AIDS Conference 2013, Kuala-Lumpur, 30 June-3 July 2013. Poster.

[10] Maman D, Masson S, Mukui I, Kirubi B, Riche B, Zeh C, et al. (2013) High incidence despite increasing coverage in Western Kenya: the Ndhiwa HIV Impact in Population Study. 17th International Conference on AIDS and STIs in Africa (ICASA), Cape Town (South Africa), 7–11 December 2013. Poster.

[11] Williams BG, Granich R, De Cock KM, Glaziou P, Sharma A, Dye C (2010) Antiretroviral therapy for tuberculosis control in nine African countries. Proc Natl Acad Sci U S A 107: 19485–19489. 1005660107 [pii]; 10.1073/pnas.1005660107 20974976 PMC2984151

[12] Pantazis N, Morrison C, Amornkul PN, Lewden C, Salata RA, Minga A, et al (2012) Differences in HIV natural history among African and non-African seroconverters in Europe and seroconverters in sub-Saharan Africa. PLoS One 7: e32369 10.1371/journal.pone.0032369PONE-D-11-21322 [pii]. 22412867 PMC3295758

[13] McKinnon LR, Nagelkerke NJ, Kaul R, Shaw SY, Capina R, Luo M, et al (2012) HIV-1 clade D is associated with increased rates of CD4 decline in a Kenyan cohort. PLoS One 7: e49797 10.1371/journal.pone.0049797PONE-D-12-20406 [pii]. 23185441 PMC3504142

[14] Cohen MS, Chen YQ, McCauley M, Gamble T, Hosseinipour MC, Kumarasamy N, et al (2011) Prevention of HIV-1 infection with early antiretroviral therapy. N Engl J Med 365: 493–505. 10.1056/NEJMoa1105243 21767103 PMC3200068

[15] Auvert B, Taljaard D, Lagarde E, Sobngwi-Tambekou J, Sitta R, Puren A (2005) Randomized, controlled intervention trial of male circumcision for reduction of HIV infection risk: the ANRS 1265 Trial. PLoS Med 2: e298 05-PLME-RA-0310R1 [pii]; 10.1371/journal.pmed.0020298 16231970 PMC1262556

[16] Bailey RC, Moses S, Parker CB, Agot K, Maclean I, Krieger JN, et al (2007) Male circumcision for HIV prevention in young men in Kisumu, Kenya: a randomised controlled trial. Lancet 369: 643–656. S0140-6736(07)60312-2 [pii]; 10.1016/S0140-6736(07)60312-2 17321310

[17] Gray RH, Kigozi G, Serwadda D, Makumbi F, Watya S, Nalugoda F, et al (2007) Male circumcision for HIV prevention in men in Rakai, Uganda: a randomised trial. Lancet 369: 657–666. S0140-6736(07)60313-4 [pii]; 10.1016/S0140-6736(07)60313-4 17321311

[18] Auvert B, Taljaard D, Rech D, Lissouba P, Singh B, Bouscaillou J, et al (2013) Association of the ANRS-12126 male circumcision project with HIV levels among men in a South African township: evaluation of effectiveness using cross-sectional surveys. PLoS Med 10: e1001509 10.1371/journal.pmed.1001509PMEDICINE-D-12-02855 [pii]. 24019763 PMC3760784

[19] Kay R (1986) A Markov model for analyzing cancer markers and disease states in survival studies. Biometrics 42: 855–865. 2434150

[20] Andersen PK, Keiding N (2002) Multi-state models for event history analysis. Stat Methods Med Res 11: 91–115. 12040698 10.1191/0962280202SM276ra

[21] Mc Callum H, Barlow N, Hone J (2001) How should pathogen transmission be modelled? TRENDS in Ecology and Evolution 16: 295–300. 11369107 10.1016/s0169-5347(01)02144-9

[22] Kenya National Bureau of Statistics (KNBS), ICF Macro (2010) Kenya Demographic and Health Survey 2008–09.

[23] van Eijk AM, Adazu K, Ofware P, Vulule J, Hamel M, Slutsker L (2008) Causes of deaths using verbal autopsy among adolescents and adults in rural western Kenya. Trop Med Int Health 13: 1314–1324. TMI2136 [pii]; 10.1111/j.1365-3156.2008.02136.x 18721187

[24] Amornkul PN, Vandenhoudt H, Nasokho P, Odhiambo F, Mwaengo D, Hightower A, et al (2009) HIV prevalence and associated risk factors among individuals aged 13–34 years in Rural Western Kenya. PLoS One 4: e6470 10.1371/journal.pone.0006470 19649242 PMC2714463

[25] Joint United Nations Programme on HIV/AIDS (UNAIDS) (2013) Global report: UNAIDS report on the global AIDS epidemic.

[26] Lodi S, Phillips A, Touloumi G, Geskus R, Meyer L, Thiebaut R, et al (2011) Time from human immunodeficiency virus seroconversion to reaching CD4+ cell count thresholds <200, <350, and <500 Cells/mm(3): assessment of need following changes in treatment guidelines. Clin Infect Dis 53: 817–825. cir494 [pii]; 10.1093/cid/cir494 21921225

[27] Wandel S, Egger M, Rangsin R, Nelson KE, Costello C, Lewden C, et al (2008) Duration from seroconversion to eligibility for antiretroviral therapy and from ART eligibility to death in adult HIV-infected patients from low and middle-income countries: collaborative analysis of prospective studies. Sex Transm Infect 84 Suppl 1: i31–i36. 84/Suppl_1/i31 [pii]; 10.1136/sti.2008.029793 PMC2569418 18647863

[28] Johnson LF, Kranzer K, Middelkoop K, Wood R (2011) A model of the impact of HIV/AIDS and antiretroviral treatment in the Masiphumelele community. Centre for Infectious Disease Epidemiology and Research Working Paper Available at URL: http://webdav.uct.ac.za/depts/epi/publications/documents/Masiphumelele%20ART%20model%202011.pdf. Accessed November 2014.

[29] Jahn A, Floyd S, Crampin AC, Mwaungulu F, Mvula H, Munthali F, et al (2008) Population-level effect of HIV on adult mortality and early evidence of reversal after introduction of antiretroviral therapy in Malawi. Lancet 371: 1603–1611. S0140-6736(08)60693-5 [pii]; 10.1016/S0140-6736(08)60693-5 18468544 PMC2387197

[30] Crampin AC, Floyd S, Glynn JR, Sibande F, Mulawa D, Nyondo A, et al (2002) Long-term follow-up of HIV-positive and HIV-negative individuals in rural Malawi. AIDS 16: 1545–1550. 12131193 10.1097/00002030-200207260-00012

[31] Zaba B, Marston M, Crampin AC, Isingo R, Biraro S, Barnighausen T, et al (2007) Age-specific mortality patterns in HIV-infected individuals: a comparative analysis of African community study data. AIDS 21 Suppl 6: S87–S96. 10.1097/01.aids.0000299415.67646.2600002030-200711006-00012 [pii]. 18032944

[32] Kasamba I, Baisley K, Mayanja BN, Maher D, Grosskurth H (2012) The impact of antiretroviral treatment on mortality trends of HIV-positive adults in rural Uganda: a longitudinal population-based study, 1999–2009. Trop Med Int Health 17: e66–e73. 10.1111/j.1365-3156.2012.02841.x 22943381 PMC3443388

[33] Alsallaq RA, Baeten JM, Celum CL, Hughes JP, Abu-Raddad LJ, Barnabas RV, et al (2013) Understanding the potential impact of a combination HIV prevention intervention in a hyper-endemic community. PLoS One 8: e54575 10.1371/journal.pone.0054575PONE-D-11-25870 [pii]. 23372738 PMC3553021

[34] Abbas UL, Glaubius R, Mubayi A, Hood G, Mellors JW (2013) Antiretroviral therapy and pre-exposure prophylaxis: combined impact on HIV transmission and drug resistance in South Africa. J Infect Dis 208: 224–234. jit150 [pii]; 10.1093/infdis/jit150 23570850 PMC3895950

[35] Cremin I, Alsallaq R, Dybul M, Piot P, Garnett G, Hallett TB (2013) The new role of antiretrovirals in combination HIV prevention: a mathematical modelling analysis. AIDS 27: 447–458. 10.1097/QAD.0b013e32835ca2dd00002030-201301280-00015 [pii]. 23296196

[36] Eaton JW, Menzies NA, Stover J, Cambiano V, Chindelevitch L, Cori A, et al (2014) Health benefits, costs, and cost-eff ectiveness of earlier eligibility for adult antiretroviral therapy and expanded treatment coverage: a combined analysis of 12 mathematical models. Lancet Glob Health 2: e23–e34. 10.1016/S2214-109X(13)70172-4 25104632

[37] Couchoud C, Dantony E, Elsensohn MH, Villar E, Ecochard R (2013) Modelling treatment trajectories to optimize the organization of renal replacement therapy and public health decision-making. Nephrol Dial Transplant 28: 2372–2382. gft204 [pii]; 10.1093/ndt/gft204 23787553

[38] Powers KA, Ghani AC, Miller WC, Hoffman IF, Pettifor AE, Kamanga G, et al (2011) The role of acute and early HIV infection in the spread of HIV and implications for transmission prevention strategies in Lilongwe, Malawi: a modelling study. Lancet 378: 256–268. S0140-6736(11)60842-8 [pii]; 10.1016/S0140-6736(11)60842-8 21684591 PMC3274419

[39] Anderson SJ, Cherutich P, Kilonzo N, Cremin I, Fecht D, Kimanga D, et al (2014) Maximising the effect of combination HIV prevention through prioritisation of the people and places in greatest need: a modelling study. Lancet 384: 249–256. S0140-6736(14)61053-9 [pii]; 10.1016/S0140-6736(14)61053-9 25042235

[40] National AIDS and STI Control Programme (NASCOP) Kenya (2009) Kenya AIDS Indicator Survey 2009: Final Report. Nairobi, NASCOP, September 2009.

[41] Shisana O, Rehle T, Simbayi LC, Zuma K, Jooste S, Zungu N, et al (2014) South African National HIV Prevalence, Incidence and Behaviour Survey, 2012 Cape Town, HSRC Press. 10.2989/16085906.2016.1153491 27002359

[42] National AIDS and STI Control Programme (NASCOP) Kenya (2014) Kenya AIDS Indicator Survey 2014: Final Report. Nairobi, NASCOP, June 2014.

[43] Bellan SE, Fiorella KJ, Melesse DY, Getz WM, Williams BG, Dushoff J (2013) Extra-couple HIV transmission in sub-Saharan Africa: a mathematical modelling study of survey data. Lancet 381: 1561–1569. S0140-6736(12)61960-6 [pii]; 10.1016/S0140-6736(12)61960-6 23391466 PMC3703831

[44] Bellan SE, Dushoff J, Galvani AP, Meyers LA (2015) Reassessment of HIV-1 acute phase infectivity: accounting for heterogeneity and study design with simulated cohorts. PLoS Med 12: e1001801 10.1371/journal.pmed.1001801PMEDICINE-D-14-01583 [pii]. 25781323 PMC4363602

[45] Nagelkerke NJ, Moses S, de Vlas SJ, Bailey RC (2007) Modelling the public health impact of male circumcision for HIV prevention in high prevalence areas in Africa. BMC Infect Dis 7: 16 1471-2334-7-16 [pii]; 10.1186/1471-2334-7-16 17355625 PMC1832203

[46] Boily MC, Bastos FI, Desai K, Masse B (2004) Changes in the transmission dynamics of the HIV epidemic after the wide-scale use of antiretroviral therapy could explain increases in sexually transmitted infections: results from mathematical models. Sex Transm Dis 31: 100–113. 10.1097/01.OLQ.0000112721.21285.A2 14743073

[47] Abbas UL, Glaubius R, Hood G, Mellors JW (2014) Antiretroviral treatment, preexposure prophylaxis, and drug resistance in sub-Saharan Africa: a consensus among mathematical models. J Infect Dis 209: 164–166. jit545 [pii]; 10.1093/infdis/jit545 24133186

[48] Kretzschmar ME, Schim van der Loeff MF, Birrell PJ, De Angelis D, Coutinho RA (2013) Prospects of elimination of HIV with test-and-treat strategy. Proc Natl Acad Sci U S A 110: 15538–15543. 1301801110 [pii]; 10.1073/pnas.1301801110 24009342 PMC3785722

[49] World Health Organization (2013) Consolidated guidelines on general HIV care and the use of antiretroviral drugs for treating and preventing HIV infection: recommendations for a public health approach. 26042326

[50] Wawer MJ, Gray RH, Sewankambo NK, Serwadda D, Li X, Laeyendecker O, et al (2005) Rates of HIV-1 transmission per coital act, by stage of HIV-1 infection, in Rakai, Uganda. J Infect Dis 191: 1403–1409. JID33445 [pii]; 10.1086/429411 15809897

[51] Granich RM, Gilks CF, Dye C, De Cock KM, Williams BG (2009) Universal voluntary HIV testing with immediate antiretroviral therapy as a strategy for elimination of HIV transmission: a mathematical model. Lancet 373: 48–57. S0140-6736(08)61697-9 [pii]; 10.1016/S0140-6736(08)61697-9 19038438

[52] Baggaley RF, Garnett GP, Ferguson NM (2006) Modelling the impact of antiretroviral use in resource-poor settings. PLoS Med 3: e124 05-PLME-RA-0204R2 [pii]; 10.1371/journal.pmed.0030124 16519553 PMC1395349

