# Supplementary material for: Estimation and Short-Term Prediction of the Course of the HIV Epidemic Using Demographic and Health Survey Methodology-Like Data
Source: PLoS One. 2015 Jun 19;10(6):e0130387. doi: 10.1371/journal.pone.0130387 (PMC4474856; doi:10.1371/journal.pone.0130387)
Supplement: S1 Text — (PDF) [file pone.0130387.s001.pdf]

Appendix

Estimation and short-term prediction of the course of the HIV epidemic using Demographic and Health Survey methodology-like data

Stéphanie Blaizot, Benjamin Riche, David Maman, Irene Mukui, Beatrice Kirubi, Jean-François Etard, René Ecochard

The model describes HIV transmission, the untreated-disease progression, and ART use in a heterosexual population. It splits the population into compartments according to sex, age, and HIV status. Infected individuals were separated into three compartments according to the CD4 cell count and the ART status: 1) Compartment I_1: untreated HIV-positive individuals with a CD4 cell count > 350 cells/mm^3; 2) Compartment I_2: untreated HIV-positive individuals with a CD4 cell count ≤ 350 cells/mm^3 (immunosuppressed individuals); and, 3) Compartment T: HIV-positive individuals under ART. An additional Compartment S was dedicated to HIV-negative (or susceptible) individuals. The model parameters are the following: i) the force of infection (λ_S); ii) the "immunosuppression rate" (λ_I); i.e., the rate at which an individual moves from > 350 to ≤ 350 CD4 cells/mm^3; iii) the "treatment rate" (λ_T) or the ART initiation rate; and, iv) the mortality rate (μ).

We describe here the methodology used to estimate the model parameters (including the data used from the survey and the inference methods used) and provide the differential equations used to predict the short-term spread of the HIV epidemic.

Estimation of the force of infection, the immunosuppression rate, and the treatment rate

Data used

We used the HIV status, the self-reported ART status, and the CD4 cell count at the time of the survey to assign each individual to one of the compartments of the model. Then, we used the individuals' histories (self-reported date of the first positive HIV test, date of the last HIV test and its result, date of ART initiation) and the CD4 cell count reduction per year to build a

working dataset with the individuals' transitions within the year preceding the survey. We reconstructed only the preceding year to minimize the recall bias and avoid making too strong assumptions about individuals' histories.

More precisely, HIV-negative individuals at the date of the survey were considered as previously HIV-negative. Depending on the date of the first positive HIV test and/or the result of the last HIV test, untreated HIV-positive individuals with CD4 cell counts > 350 cells/mm^3 (Compartment I_1) were considered to have been already HIV-positive (positive test result) or HIV-negative (negative test result) one year before the survey. In untreated HIV-positive individuals with CD4 cell counts ≤ 350 cells/mm^3 at the moment of the survey (Compartment I_2), the CD4 cell count one year before was obtained by applying a 15% CD4 cell count reduction per year [1]. Depending on the date of ART initiation, treated individuals were considered to have been already under treatment one year preceding the survey or untreated HIV-positive with CD4 cell counts ≤ 350 cells/mm^3 one year preceding the survey.

Logical or probabilistic rules were applied when the retrospective information was incomplete or lacking (6.3% and 1.2% of all cases, respectively). The logical rules consisted in the following assumptions: i) HIV-positive individuals under a treatment initiated within the previous year were considered to have been HIV-positive one year preceding the survey; ii) untreated HIV-positive individuals with low CD4 cell counts were considered to have been already HIV-positive one year preceding the survey; iii) untreated HIV-positive individuals with high CD4 cell counts but no information about previous testing were considered to have been HIV-positive at least one month preceding the survey.

A sensitivity analysis about the assumption of the CD4 cell count decline was performed assuming a 10% decline per year [2] as well as a 20% decline per year [3].

Inference methods

The individuals' states and the times spent in each state make it possible to estimate the model parameters using a statistical method based on the likelihood decomposition [4,5]. In this method, each state is considered sequentially and the likelihood of the complete model is decomposed into several conditionally independent likelihoods. This decomposition is feasible whenever there are no interactions between the individual courses. Here, the probability of infection for an uninfected individual depends on HIV prevalence. At the estimation step, the period being about one year, the change in prevalence was nearly negligible. Thus, at this step, the force of infection was not split into prevalence and a transmission coefficient.

For example, we consider a sub-model composed of an initial state C_1 and other states $C_2 \ldots C_n$. Let λ_{1j} be the transition rate between C_1 and another state C_j to estimate. Over a given period, let E_1 be the time spent at risk in C_1, N_{1j} the number of transitions observed between C_1 and C_j. Then, N_{1j} can be modeled as a realization of a Poisson process of mean μ_{1j}:

$$\mu_{1j} = E_1 \cdot \lambda_{1j}$$

The conditional log-likelihood of a Poisson distribution is then:

$$LL = N_{1j} \cdot \log(\mu_{1j}) - \mu_{1j}$$

This likelihood was maximized using the Newton-Raphson option of PROC NLP of SAS 9.2 software. The confidence intervals were calculated with Wald method.

We estimated the model parameters in men and women, in three age groups (15-24, 25-34, and 35-59 years), and using a stratification by six “Divisions” (or residence areas).

Estimation of the mortality rates

The NHIPS survey reported only 37 deaths. This information was not sufficient to estimate accurately the mortality rates with the likelihood decomposition method. Thus, to estimate non-AIDS-related and AIDS-related mortality rates, we used Poisson regression with the overall mortality rates from the Kenyan DHS (KDHS) [6] and external data on the proportion of AIDS-related deaths [7,8]. We noticed that the 37 deaths were compatible with the mortality rates estimated here.

The number of deaths, d, given the time spent at risk of death, E, can be described as a Poisson distribution. We assumed that S, I_1, and T individuals had the same risks of death, that I_2 individuals had an additional risk of death due to AIDS, and that all had the same risk of dying from other causes than AIDS. All the subjects were thus exposed to the overall mortality rate μ_S related to other causes of death than AIDS. Moreover, a proportion p_{Ndhiwa} was exposed to an AIDS-related mortality rate δ. The Poisson distribution that describes the number of deaths can be further refined: the expected number of deaths within the population is the sum of the number of deaths related to AIDS ($p_{Ndhiwa} \cdot E \cdot \delta$) and the number of deaths related to other causes ($E \cdot \mu_S$). The parameter of the Poisson distribution is the expected number of deaths under this assumption; thus:

$$d \sim Poisson\left(p_{Ndhiwa} \cdot E \cdot \delta + E \cdot \mu_S\right)$$

Rewriting this equation and using an external estimation of the ratio between AIDS-related and non-AIDS-related mortality rates from [7], denoted $r*$:

$$d \sim Poisson\left(E \cdot \mu_S \cdot \left[p_{Ndhiwa} \cdot r* + 1\right]\right)$$

We then estimated the mortality rates using:

$\log(\mathrm{E}(d)) = \log\left(E \cdot \left[p_{Ndhiwa} \cdot r* + 1\right]\right)$ for S, I_1, and T individuals

and $\log(\mathrm{E}(d)) = \log\left(E \cdot \left[\dfrac{p_{Ndhiwa} \cdot r* + 1}{r* + 1}\right]\right)$ for I_2 individuals.

Prediction

The mathematical model that provides short-term predictions of the course of the HIV epidemic was formulated as a system of sex- and age-specific (*a* = 15, ..., 59 years) differential equations:

$$\begin{cases} \dfrac{dS_{Sex,a}}{dt} = -\lambda_{S,Sex,a}\ S_{Sex,a} - \mu_{S,Sex,a}\ S_{Sex,a} - \nu\ S_{Sex,a} + \nu\ S_{Sex,a-1} \\ \dfrac{dI_{1Sex,a}}{dt} = \lambda_{S,Sex,a}\ S_{Sex,a} - \left(\mu_{I_1,Sex,a} + \lambda_{I,Sex,a}\right) I_{1Sex,a} - \nu\ I_{1Sex,a} + \nu\ I_{1Sex,a-1} \\ \dfrac{dI_{2Sex,a}}{dt} = \lambda_{I,Sex,a}\ I_{1Sex,a} - \left(\mu_{I_2,Sex,a} + \lambda_{T,Sex,a}\right) I_{2Sex,a} - \nu\ I_{2Sex,a} + \nu\ I_{2Sex,a-1} \\ \dfrac{dT_{Sex,a}}{dt} = \lambda_{T,Sex,a}\ I_{2Sex,a} - \mu_{T,Sex,a}\ T_{Sex,a} - \nu\ T_{Sex,a} + \nu\ T_{Sex,a-1} \end{cases}$$

Ageing was considered through the last two terms of each equation; ν being the rate at which an individual moves from one age class to another. For the first age class (15 years), ageing was taken into account as follows: at one year intervals, a fixed number of individuals were shifted into compartment S.

The force of infection is frequency-dependent [9]; it included HIV prevalence in the opposite sex weighted by the infectiousness of HIV-positive individuals. This infectiousness (probability of transmitting the virus) depends on the use of ART. This force of infection may then be written as follows:

$$\lambda_{S,Sex,a} = \tilde{\beta}_{Sex,a} \left(\frac{I_{1OppositeSex} + I_{2OppositeSex} + \varepsilon T_{OppositeSex}}{N_{OppositeSex}} \right) \zeta_{Sex,a}$$

where ε is the reduction of infectiousness due to ART and $\tilde{\beta}_{Sex,a}$ the transmission parameter. $\zeta_{Sex,a} = (1-\varphi_{Sex,a}) + \gamma\,\varphi_{Sex,a}$ allows for the possibility that some individuals, a proportion φ, have different susceptibilities (reduced by a given value γ) due, for example, to circumcision in men.

Code:

```
%macro macroPM (input);
   proc model data=&input mintimestep=1.0e-23;
      endog NbSFemale15-NbSFemale59 NbSMale15-NbSMale59
            NbVFemale15-NbVFemale59 NbVMale15-NbVMale59
            NbAFemale15-NbAFemale59 NbAMale15-NbAMale59
            NbTFemale15-NbTFemale59 NbTMale15-NbTMale59 ;

      %do j=1 %to 2; /* Loop on sex */
         %if &j.=2 %then %do; %let s=Female; %let s2=Male; %end;
         %if &j.=1 %then %do; %let s=Male; %let s2=Female; %end;

         /*-------------------------------------------------------------*/
         /*Calculation of the prevalence by sex */
         %do a2=15 %to 59; /* Loop on age */
            Inf_num&s2.&a2.=NbV&s2.&a2. + NbA&s2.&a2. +( NbT&s2.&a2.
                            * &param_ARV_reduc );
            Inf_denom&s2.&a2.=NbV&s2.&a2. + NbA&s2.&a2. + NbT&s2.&a2.+ NbS&s2.&a2.;
         %end;

         Inf_num&s2.=sum(of Inf_num&s2.15-Inf_num&s2.59);
         Inf_denom&s2.=sum(of Inf_denom&s2.15-Inf_denom&s2.59);
         /*-------------------------------------------------------------*/

         %do a=15 %to 59; /* Loop on age */

            %let a_1 = %eval(&a.-1);

            /* Loop no 1 : 15 yrs old */
            %if &a.=15 %then %do ;

               /*dS/dt */
```

```
            dert.NbS&s.&a. =  ( - muSD&s.&a. * NbS&s.&a. -
              param_Beta&s.&a. * NbS&s.&a. * ((1 - PropSuscProtege&s.&a.) +
              PropSuscProtege&s.&a. * SuscAvecProtec&s.&a.) * (Inf_num&s2. /
              Inf_denom&s2.) + (1/12)*nu&s.&a. - (1/12)*NbS&s.&a. );

            /*dI1/dt */
            dert.NbV&s.&a. = ( - lambdaVA&s.&a. * NbV&s.&a.
              - muVD&s.&a. * NbV&s.&a. + param_Beta&s.&a. * NbS&s.&a. *
              ((1 - PropSuscProtege&s.&a.) + PropSuscProtege&s.&a. *
              SuscAvecProtec&s.&a.) * (Inf_num&s2./ Inf_denom&s2.) -
              (1/12)*NbV&s.&a.);

            /*dI2/dt*/
            dert.NbA&s.&a. = ( lambdaVA&s.&a. * NbV&s.&a. -
              lambdaAT&s.&a. * NbA&s.&a. -
              muAD&s.&a. * NbA&s.&a. - (1/12)*NbA&s.&a. );

            /*dT/dt */
            dert.NbT&s.&a. = ( lambdaAT&s.&a. * NbA&s.&a.
              - muTD&s.&a. * NbT&s.&a.  - (1/12)*NbT&s.&a. );
         %end;
         /* End Loop 1 */

         /* Loop no 2 :  > 15 yrs old */
         %if &a. ne 15 %then %do;

            /*dS/dt */
            dert.NbS&s.&a. = ( - muSD&s.&a. * NbS&s.&a. -
               param_Beta&s.&a. * NbS&s.&a. * ((1 - PropSuscProtege&s.&a.)
               + PropSuscProtege&s.&a. * SuscAvecProtec&s.&a.) * (Inf_num&s2. /
               Inf_denom&s2.) + (1/12)*NbS&s.&a_1. - (1/12)*NbS&s.&a. );

            /*dI1/dt */
            dert.NbV&s.&a. = ( - lambdaVA&s.&a. * NbV&s.&a. -
               muVD&s.&a. * NbV&s.&a. + param_Beta&s.&a. * NbS&s.&a. *
               ((1 - PropSuscProtege&s.&a.) + PropSuscProtege&s.&a. *
               SuscAvecProtec&s.&a.) * (Inf_num&s2./ Inf_denom&s2.) +
               (1/12)*NbV&s.&a_1. - (1/12)*NbV&s.&a. );

            /*dI2/dt*/
            dert.NbA&s.&a. = (lambdaVA&s.&a. * NbV&s.&a. -
               lambdaAT&s.&a. * NbA&s.&a. -
               muAD&s.&a. * NbA&s.&a. + (1/12)*NbA&s.&a_1. - (1/12)*NbA&s.&a. );

            /*dT/dt */
            dert.NbT&s.&a. = (lambdaAT&s.&a. * NbA&s.&a. -
               muTD&s.&a. * NbT&s.&a. + (1/12)*NbT&s.&a_1. - (1/12)*NbT&s.&a.) ;

         %end;
         /* End loop 2 */

      %end;
   %end;
   /*-------------------------------------------------------------------------*/

   solve  NbSFemale15-NbSFemale59 NbSMale15-NbSMale59
          NbVFemale15-NbVFemale59 NbVMale15-NbVMale59
          NbAFemale15-NbAFemale59 NbAMale15-NbAMale59
          NbTFemale15-NbTFemale59 NbTMale15-NbTMale59 /
          dynamic time=month out=ProcModel_Pred outpredict converge=0.0000001;
   run; quit;
%mend;
```

References

1. Williams BG, Granich R, De Cock KM, Glaziou P, Sharma A, Dye C (2010) Antiretroviral therapy for tuberculosis control in nine African countries. Proc Natl Acad Sci U S A 107: 19485-19489. 1005660107 [pii];10.1073/pnas.1005660107 [doi].

2. Pantazis N, Morrison C, Amornkul PN, Lewden C, Salata RA, Minga A, Chipato T, Jaffe H, Lakhi S, Karita E, Porter K, Meyer L, Touloumi G (2012) Differences in HIV natural history among African and non-African seroconverters in Europe and seroconverters in sub-Saharan Africa. PLoS One 7: e32369. 10.1371/journal.pone.0032369 [doi];PONE-D-11-21322 [pii].

3. McKinnon LR, Nagelkerke NJ, Kaul R, Shaw SY, Capina R, Luo M, Kariri A, Apidi W, Kimani M, Wachihi C, Jaoko W, Anzala AO, Kimani J, Ball TB, Plummer FA (2012) HIV-1 clade D is associated with increased rates of CD4 decline in a Kenyan cohort. PLoS One 7: e49797. 10.1371/journal.pone.0049797 [doi];PONE-D-12-20406 [pii].

4. Kay R (1986) A Markov model for analyzing cancer markers and disease states in survival studies. Biometrics 42: 855-865.

5. Andersen PK, Keiding N (2002) Multi-state models for event history analysis. Stat Methods Med Res 11: 91-115.

6. Kenya National Bureau of Statistics (KNBS), ICF Macro (2010) Kenya Demographic and Health Survey 2008-09.

7. van Eijk AM, Adazu K, Ofware P, Vulule J, Hamel M, Slutsker L (2008) Causes of deaths using verbal autopsy among adolescents and adults in rural western Kenya. Trop Med Int Health 13: 1314-1324. TMI2136 [pii];10.1111/j.1365-3156.2008.02136.x [doi].

8. Amornkul PN, Vandenhoudt H, Nasokho P, Odhiambo F, Mwaengo D, Hightower A, Buve A, Misore A, Vulule J, Vitek C, Glynn J, Greenberg A, Slutsker L, De Cock KM (2009) HIV prevalence and associated risk factors among individuals aged 13-34 years in Rural Western Kenya. PLoS One 4: e6470. 10.1371/journal.pone.0006470 [doi].

9. Mc Callum H, Barlow N, Hone J (2001) How should pathogen transmission be modelled? Trends Ecol Evol 16: 295-300.
